# Amelioration of gentamicin-induced acute kidney injury by trifluoperazine: in vivo mechanistic insights

**DOI:** 10.1038/s41598-026-47243-w

**Published:** 2026-04-20

**Authors:** Ahmed E. Goda, Nereen A. Almosilhy, Nageh A. El-Mahdy

**Affiliations:** Department of Pharmacology and Toxicology, Faculty of Pharmacy, Tanta University, Tanta, 31527 Egypt

**Keywords:** Trifluoperazine, Gentamicin, Acute kidney injury, Oxidative stress, Autophagy, p-ERK1/2, NF-κB/NLRP3 inflammasomes, Biochemistry, Diseases, Drug discovery, Medical research, Molecular biology, Nephrology

## Abstract

Acute kidney injury (AKI) is a serious risk associated with gentamicin. Therefore, the current study investigated whether pretreatment with the antipsychotic trifluoperazine could provide protective effects. Assessment of serum creatinine and blood urea nitrogen, in addition to histopathology analysis, demonstrated that trifluoperazine effectively protected the kidneys from functional and structural abnormalities induced by gentamicin. Several signaling pathways that were modulated by drug treatments were investigated in the kidney and showed that trifluoperazine mitigated gentamicin-triggered oxidative stress evidenced by the preservation of SOD activity, reduction of MDA levels, and inhibition of the transcription of stress-responsive genes, *Chop* and *Ho-1*. Moreover, trifluoperazine abrogated the increased protein levels of the p65 subunit of NF-κB and suppressed the transcription of NF-κB target genes, *IL-1β, TNF-α,* and *Tsp-1*, highlighting the inhibition of the aberrantly activated NF-κB signaling. Further, trifluoperazine attenuated the increased ERK1/2 phosphorylation by gentamicin. Notably, p62 and LC3B protein levels indicated that trifluoperazine alleviated the impaired autophagy by gentamicin. Additionally, NLRP3 and IL-1β protein levels suggested that trifluoperazine mitigated gentamicin-induced NLRP3 inflammasomes activation. Finally, trifluoperazine effectively suppressed apoptosis induction by gentamicin. In conclusion, our study provided the first in vivo preclinical evidence supporting further investigation of the potential of trifluoperazine to mitigate gentamicin-induced AKI.

**Supplementary Information:**

The online version contains supplementary material available at 10.1038/s41598-026-47243-w.

## Introduction

Severe bacterial infections pose a significant challenge in critical care medicine, primarily due to antimicrobial resistance and the dose-limiting side effects of current therapies. The declining rate of new antimicrobial development further exacerbates the problem^1^. Gentamicin, an aminoglycoside antibiotic, is preferred for severe gram-negative infections in intensive care due to its rapid bactericidal activity, low resistance rates, convenient administration, low cost, and synergistic effects with other antibiotics^2,3^. However, nephrotoxicity and ototoxicity are significant adverse effects that complicate treatment outcomes. Up to 20% of critically ill sepsis patients receiving gentamicin for over 72 h develop acute kidney injury (AKI), marked by renal dysfunction that may progress to chronic kidney disease^2,4–6^.

Gentamicin induces AKI through the excessive generation of oxidative stress in renal cells, primarily by enhancing mitochondrial production of reactive oxygen species (ROS) and reducing the activity of superoxide dismutase (SOD), a key antioxidant enzyme. This oxidative stress damages cellular biomolecules and activates signaling pathways, including nuclear factor-κB (NF-κB), extracellular signal-regulated kinase (ERK), and NOD-like receptor family, pyrin domain containing 3 (NLRP3) inflammasomes, while disrupting autophagy, a vital cellular degradation process^7–12^. These alterations ultimately contribute to AKI pathogenesis. Consequently, identifying a therapeutic strategy to mitigate gentamicin-induced AKI is clinically crucial.

Trifluoperazine, originally an antipsychotic, has recently gained attention for its pleiotropic effects on both normal and cancer cells, suggesting potential for repurposing in novel therapeutic indications^13–18^.

Through distinct mechanisms, trifluoperazine has demonstrated protective effects on various organs and tissues against deleterious insults, primarily as a potent calmodulin inhibitor^19^. Trifluoperazine’s blockade of calmodulin has been shown to protect against oxidative stress and apoptosis^20,21^. Additionally, it exhibited anti-inflammatory activity in vivo^22–24^ and promoted cytoprotective autophagy^15–17,25^. For instance, trifluoperazine conferred significant cardioprotection against doxorubicin-induced cardiotoxicity^22^ and provided neuroprotection in conditions such as Huntington’s disease, acute stroke-related cerebral edema, and traumatic brain injury^15–17^. These protective effects are attributed to preserved autophagy, facilitating clearance of misfolded protein aggregates, and suppression of apoptosis, inflammation, and cerebral edema via inhibition of aquaporin-4 accumulation on brain cell surfaces^15,17^.

Given trifluoperazine’s promising protective potential, this in vivo study investigated whether a clinically relevant dosing regimen of trifluoperazine could extend gentamicin treatment beyond 72 h while preventing AKI. Additionally, the underlying protective signaling pathways were also examined.

## Materials and methods

### Drugs

Gentamicin sulfate was generously obtained from Memphis Pharmaceutical & Chemical Industries Company (Egypt). Trifluoperazine was generously provided by Kahira Pharmaceuticals & Chemical Industries (Egypt). Both drugs were dissolved in sterile saline immediately before daily injections to the mice.

### Animals

A total of 48 adult male Swiss albino mice weighing between 21 and 26 g (8 to 12 weeks old) were sourced from the National Research Center in Giza, Egypt. Before commencing drug treatment, mice were allowed to acclimatize for one week with free access to standard pellet chow and water. Animal experiments were conducted following the approval of the Research Ethics Committee of the Faculty of Pharmacy, Tanta University, EGYPT (TP/RE/04/22M-0015). All animal experiments were carried out in accordance with the U.K. Animals (Scientific Procedures) Act, 1986, and associated guidelines, EU Directive 2010/63/EU for animal experiments, and the National Research Council’s Guide for the Care and Use of Laboratory Animals. Furthermore, reporting of animal testing experiments complied with the ARRIVE guidelines.

### In vivo induction of AKI by gentamicin

Mice were randomly assigned to the following four groups (12 mice each):

I. Control group received sterile saline via intraperitoneal (I.P.) injection once daily for 15 consecutive days.
II. Gentamicin group received a single daily I.P. injection of 2.35 mg/mouse from a 10 mg/mL gentamicin solution (100 mg/kg/day)^26^ for 8 consecutive days.
III. Trifluoperazine group received a single daily I.P. injection of 0.035 mg/mouse from a 0.15 mg/mL trifluoperazine solution (1.5 mg/kg/day)^27^ for 15 consecutive days.
IV. Trifluoperazine/gentamicin group received I.P. injections of 0.035 mg/mouse from a 0.15 mg/mL trifluoperazine solution (1.5 mg/kg/day) for 7 days, followed by concurrent treatment with 2.35 mg/mouse from a 10 mg/mL gentamicin solution (100 mg/kg/day) for additional 8 days, with a 3-h interval between doses.

#### Dosing regimen and its clinical relevance

The doses of trifluoperazine and gentamicin utilized in this study are clinically relevant based on the principles of dose extrapolation described in the FDA guidelines for converting animal doses to equivalent human doses^28^. The trifluoperazine mouse dose of 1.5 mg/kg/day is equivalent to a human dose of 0.12 mg/kg/day or a total daily dose of 7.3 mg. The recommended daily dose for patients with schizophrenia usually ranges from 12 to 50 mg^29^. As for gentamicin, a mouse dose of 100 mg/kg/day is equivalent to a human dose of 8 mg/kg/day, which is the recommended initial dose for critically ill patients with gram-negative sepsis^2^.

Twenty-four hours after the final gentamicin injection, mice were euthanized by decapitation under ketamine anesthesia, with one mouse euthanized at a time. The blood was collected, and the kidneys were harvested. For each mouse, the right kidney was preserved in a 10% neutral buffered formalin solution (HT501128-4L, Sigma-Aldrich) for subsequent histopathological and immunohistochemical examinations, while the left kidney was stored at -80℃ for further biochemical analyses. Serum samples of all mice were stored at -80℃.

### Histopathological examination of the kidney

Formalin-fixed 5 kidneys from each group were processed for hematoxylin and eosin (ab245880, Abcam, USA) staining and scored based on the EGTI histological scoring system, assessing tubular, endothelial, interstitial, and glomerular renal tissue damage in five high-power fields for each group as previously described^30,31^.

### Serum biochemical analyses for kidney function

Kidney function tests were conducted using serum samples from mice in each group to measure blood urea nitrogen (BUN, n = 5) and creatinine (n = 10) levels spectrophotometrically using commercially available kits (UR-2110 and CR1250, respectively; Bio-diagnostic, Egypt) according to the manufacturer’s instructions.

### Measurement of lipid peroxidation levels in the kidneys

Malondialdehyde (MDA), a well-known marker for oxidative stress-mediated lipid peroxidation, was assessed in 8 kidneys from each group using a commercially available kit (MD 25 29, Bio-diagnostic, Egypt) according to the manufacturer’s instructions.

### Measurement of superoxide dismutase (SOD) enzyme activity in the kidneys

Superoxide dismutase enzyme activity was measured in 10 kidneys from each group using a commercially available kit (SD 25 21, Bio-diagnostic, Egypt) according to the manufacturer’s instructions.

### Immunohistochemical investigation.

Immunohistochemical staining was done as previously described^32^. Five kidney sections from each group were stained with the following primary antibodies at 1:100 dilution, anti-caspase 3 (C# PA5-77,887, Invitrogen, USA), anti-NF-ĸB-p65 (F-6, sc-8008, Santa Cruz, USA), Anti-LC3B (#ab48394, Abcam, USA), anti-p62 (#R31058, NSJ Bioreagents, USA), anti-phospho-ERK1/2 (Thr202, Tyr204) (#14–9109-82, Invitrogen, USA), and anti-NLRP3 inflammasome (#NBP2-12,446, Novus Biologicals, USA). Secondary antibodies used were HRP-labelled anti-rabbit antibody (#K4003) and anti-mouse antibody (#K3468) obtained from Envision™ + System (Dako, Agilent Technologies, USA). The staining intensity was assessed and presented as a percentage of positive expression in a total of 1000 cells per 5 high power fields (HPF) for NF-ĸB-p65, NLRP3, caspase 3, and p-ERK1/2, whereas the immunostaining intensity for LC3B and p62 was determined through the area of positive expression using Image J analysis software (NIH, USA) (http://imagejnih.gov/ij/).

### Enzyme-linked immunosorbent assay (ELISA)

Levels of the interleukin-1 beta (IL-1β) were assessed in 3 kidney homogenates from each group, using a commercially available ELISA kit (Cusabio-E08054m, USA), complying with the manufacturer’s instructions.

### Analysis of gene expression via semiquantitative RT-PCR

To detect early changes in gene expression in the kidneys of treated mice by semiquantitative RT-PCR, the following in vivo study was conducted (3 mice/group):

I. Control group (saline).
II. Gentamicin group (100 mg/kg/day)^26^.
III. Trifluoperazine group (1.5 mg/kg/day)^27^.
IV. Trifluoperazine (1.5 mg/kg/day) plus gentamicin (100 mg/kg/day) group.

Mice received either a single daily I.P. injection of 0.035 mg/mouse from a 0.15 mg/mL trifluoperazine solution; a single daily I.P. injection of 2.35 mg/mouse from a 10 mg/mL gentamicin solution in the induction group; or trifluoperazine pretreatment followed by gentamicin administration with a three-hour interval between doses. After 24 h, mice were euthanized by decapitation under ketamine anesthesia, and their kidneys were dissected, snap-frozen in liquid nitrogen, and processed for RNA extraction, cDNA synthesis, and PCR analysis as previously mentioned^22^. The relative expression levels of *Ho-1*, *Chop*, *TNF-α, IL-1β, Tsp-1,* and *Trp53 mRNA* were evaluated using primer sequences previously reported^33–38^. Band intensities were quantified relative to that of the *18S rRNA* housekeeping gene^39^, using the Image J program (http://imagejnih.gov/ij/).

### Western blotting

Western blotting was performed as previously described^40^ on kidney tissues that were homogenized in ice-cold 2 × Laemmli buffer. The primary antibodies used were SQSTM1/p62 (Cell Signaling Technology, USA, Cat #5114) and β-actin (Cat #4967; Cell Signaling Technology, USA). The secondary antibody used was goat anti-rabbit IgG-HRP conjugate (BioRad, USA, Cat# 1706515). The Enhanced Chemiluminescent kit used was UltraScence Pico Plus Western Substrate (BIO-HELIX Co. Ltd, TAIWAN, Cat# CCH321-B100ML). Images of visualized protein bands were captured using Azure 280 Imaging Systems (Azure Biosystems, USA).

### Statistical analysis

Data were presented as mean ± standard deviation (S.D.). Multiple comparisons among different groups were evaluated via one-way analysis of variance (ANOVA) followed by Tukey–Kramer post hoc test. On the other hand, the values of histopathological scoring expressed as median (95% CI, min–max) were analyzed using the non-parametric Kruskal–Wallis’ test, followed by Dunn’s post hoc test for Pairwise Comparisons. Statistically significant differences were considered at *p* < 0.05 using the SPSS V22.0 statistics software package (IBM, USA).

## Results

### Trifluoperazine attenuates gentamicin-induced renal dysfunction

**Fig. 1:**
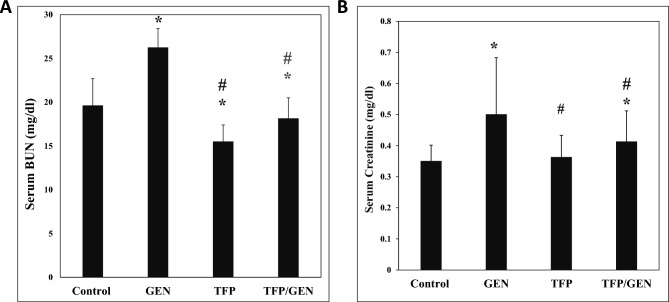
Trifluoperazine pretreatment mitigates gentamicin-induced kidney injury. Biochemical analysis of (**A**) Serum Blood Urea Nitrogen (BUN, n = 5), (**B**) Serum Creatinine (n = 10) in different groups. Data are presented as the mean ± SD. ^*^ Denotes statistically significant differences from control group. ^#^ Denotes statistically significant differences from gentamicin group. GEN, Gentamicin; TFP/GEN, Trifluoperazine and Gentamicin; TFP, Trifluoperazine.

﻿﻿﻿﻿﻿﻿To investigate the protective effect of trifluoperazine against gentamicin-induced AKI, serum levels of BUN and creatinine were measured. Results showed that gentamicin-treated mice exhibited marked renal impairment, with BUN and creatinine levels increasing by 36% and 56%, respectively, compared to controls (Fig. 1). In contrast, mice receiving trifluoperazine alone showed no abnormalities in kidney functions (Fig. 1). Interestingly, in the trifluoperazine-pretreated group﻿ prior to gentamic﻿in, BUN levels were not elevated, and creatinine levels were only 21% lower compared t﻿o﻿ the gentamicin group (Fig. ﻿﻿1). These findings suggest that trifluoperazine could significantly alleviate gentamicin-induced renal dysfunction.﻿﻿

### Trifluoperazine protects against gentamicin-induced structural kidney damage

**Fig. 2:**
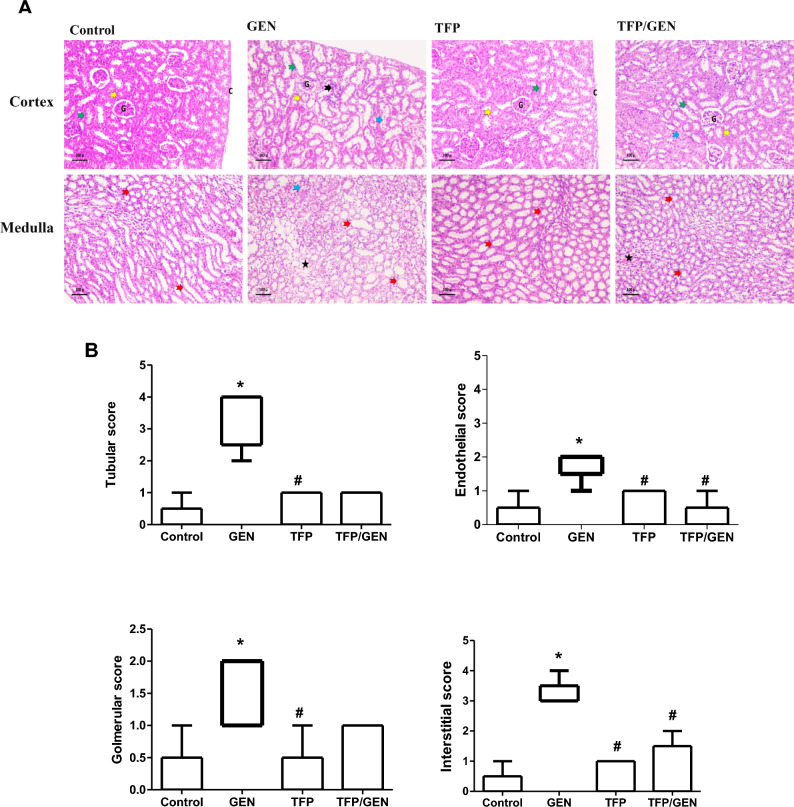
Pretreatment with trifluoperazine attenuated gentamicin-induced renal structural changes. (**A**) Representative photomicrographs of H&E-stained kidney sections from different groups (n = 5). Magnification: × 200. Bar = 50 µm. Intact capsule (C), Glomeruli (G), *Yellow arrowhead:* proximal convoluted tubule, *green arrowhead:* distal convoluted tubules, *red arrowhead:* thick ascending and descending segments of loop of Henle and collecting ducts, *blue arrowheads:* vacuolar degeneration of tubules, *black arrowhead:* focal inflammatory infiltration, *Black star:* widening and hemorrhage of interstitial tissue. (**B**) EGTI histological scoring of mice renal sections from different groups (n = 5): assessment of tubular, endothelial, glomerular, and interstitial tissue damage, data are expressed using a whisker box plot with median (max–min; n = 5 per group), then Kruskal–Wallis test followed by Dunn’s as a post-hoc test for Pairwise Comparisons. * Denotes statistically significant differences from the control group. # Denotes statistically significant differences from the gentamicin group. GEN, Gentamicin; TFP/GEN, Trifluoperazine and Gentamicin; TFP, Trifluoperazine.

To determine whether trifluoperazine’s renoprotective effects extended to tissue structure, histopathological examination was conducted. In gentamicin-treated mice, the renal cortex and medulla displayed marked architectural disruption. Cortical sections showed retracted glomeruli, loss of the brush border in proximal and distal convoluted tubules, tubular dilation, and vacuolar degeneration, along with focal inflammatory infiltration (Fig. 2A). Similarly, medullary sections revealed dilated thick segments of the loop of Henle and collecting ducts, vacuolar tubular degeneration, interstitial widening, and hemorrhage (Fig. 2A).

Conversely, mice pretreated with trifluoperazine showed significant preservation of renal histology. The renal cortex retained normal glomerular structure and intact proximal and distal convoluted tubules, with minimal tubular damage and interstitial changes (Fig. 2A). Medullary sections also showed maintained structural integrity, with preserved segments of the loop of Henle and minimal focal hemorrhage in the interstitial tissue (Fig. 2A). Kidneys from mice receiving trifluoperazine alone were histologically similar to those of untreated controls in both cortex and medulla (Fig. 2A).

To evaluate renal structural alterations, the endothelial, glomerular, tubular, and interstitial (EGTI) histological scoring system was used^31^. In the gentamicin-treated group, pronounced tubular damage was observed, with a median score of 4 (Fig. 2B), reflecting severe injury characterized by extensive brush border loss, thickened basal membranes, inflammation, and necrosis affecting up to 60% of tubular cells. On the other hand, pretreatment with trifluoperazine effectively reduced necrosis and inflammation compared to the gentamicin group, resulting in a lower median score of 1 (Fig. 2B). The trifluoperazine monotherapy group showed undetectable tubular damage, with a median score of zero, like the control group, where the basal membrane remained largely intact (Fig. 2B).

Glomerular damage assessment revealed a median score of 2 in the gentamicin-treated group, indicating notable pathological alterations such as Bowman’s capsule thickening and glomerular tuft retraction (Fig. 2B). In contrast, the trifluoperazine/gentamicin group had a median score of zero, with only mild Bowman’s capsule thickening. Similarly, the trifluoperazine monotherapy group showed a median damage score of zero, mirroring the control group (Fig. 2B).

Gentamicin treatment significantly increased endothelial damage, with a median score of 2, reflecting marked swelling and disruption of endothelial cells (Fig. 2B). Conversely, co-treatment with trifluoperazine reduced these changes, showing only mild swelling and no significant disruption or endothelial loss, with a median score of zero (Fig. 2B). Trifluoperazine monotherapy group also showed a median score of zero, similar to the control group (Fig. 2B).

Considering interstitial damage, the gentamicin-treated group exhibited significant injury, with a median score of 3, reflecting severe widening and moderate hemorrhage in the interstitial tissue (Fig. 2B). Alternatively, the trifluoperazine pretreatment group had a median score of zero, showing only mild widening (Fig. 2B). Similarly, the trifluoperazine monotherapy group also had a median score of zero, comparable to the control group (Fig. 2B).

These findings collectively indicate that trifluoperazine efficiently attenuates gentamicin-induced AKI, preserving both renal structure and function.

### Trifluoperazine mitigates oxidative stress induced by gentamicin

**Fig. 3:**
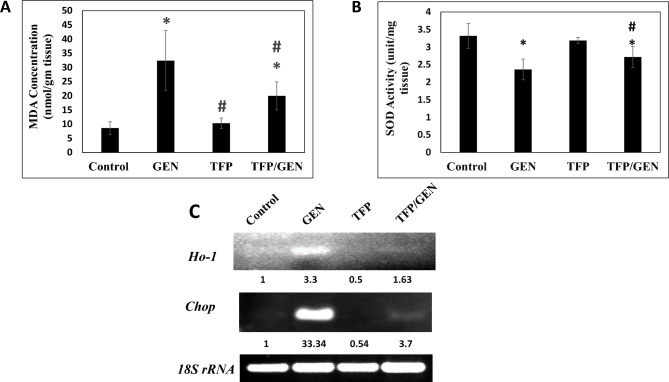
Trifluoperazine suppressed gentamicin-induced renal oxidative stress. Biochemical analysis of (**A**) MDA levels (n = 8) and (**B**) SOD activity (n = 10) in different groups. Data is presented as mean ± SD. * Denotes statistically significant differences from control group. # Denotes statistically significant differences from gentamicin group. MDA, malondialdehyde; SOD, superoxide dismutase; GEN, Gentamicin;TFP/GEN, Trifluoperazine and Gentamicin;TFP, Trifluoperazine. (**C**) Representative agarose gel electrophoresis and band intensities for PCR analysis of mRNA expression levels of *Ho-1*, *Chop,* and *18S rRNA* housekeeping control.

Given that gentamicin is a known inducer of oxidative stress^12^, MDA levels were measured as an indicator of lipid peroxidation. Gentamicin treatment led to a 3.7-fold increase in MDA levels in mice kidney tissue compared to the control (Fig. 3A). In comparison, MDA levels in the trifluoperazine monotherapy group were similar to controls. Notably, in the trifluoperazine/gentamicin group, MDA levels were only 2.3-fold higher than control, representing a 39% reduction compared to gentamicin alone (Fig. 3A). To support these findings, SOD activity was also assessed in the kidney tissue. Our findings show that gentamicin reduce SOD activity by 27% compared to the control, whereas the trifluoperazine monotherapy group showed no change (Fig. 3B). The pretreated group exhibited an 18% reduction in SOD activity compared to control (Fig. 3B). Together, these results suggest that trifluoperazine significantly ameliorates gentamicin-induced oxidative stress in the kidneys.

In view of the complex role of *Chop* in cellular responses to oxidative stress^41^. We examined the early effects of treatments on *Chop* gene expression. Gentamicin treatment induced a 33-fold upregulation of *Chop* mRNA relative to the control (Fig. 3C). Pretreatment with trifluoperazine reduced gentamicin upregulation by 89% relative to the gentamicin group (Fig. 3C). Interestingly, trifluoperazine monotherapy also decreased *Chop* mRNA levels by 46% compared to control (Fig. 3C).

The *Ho-1* gene is known to be upregulated in oxidative stress-induced AKI^42^ To assess the early impact of treatments, we evaluated *Ho-1* mRNA expression. Our data showed a substantial increase in *Ho-1* transcription, with gentamicin elevating levels by approximately threefold compared to the control (Fig. 3C). Notably, pretreatment with trifluoperazine reduced this upregulation by 51%, while trifluoperazine alone led to a 50% decrease in *Ho-1* mRNA levels relative to control (Fig. 3C). These results support the potential of trifluoperazine to mitigate gentamicin-induced oxidative stress.

### Trifluoperazine preserved autophagy in the kidneys despite gentamicin treatment

**Fig. 4:**
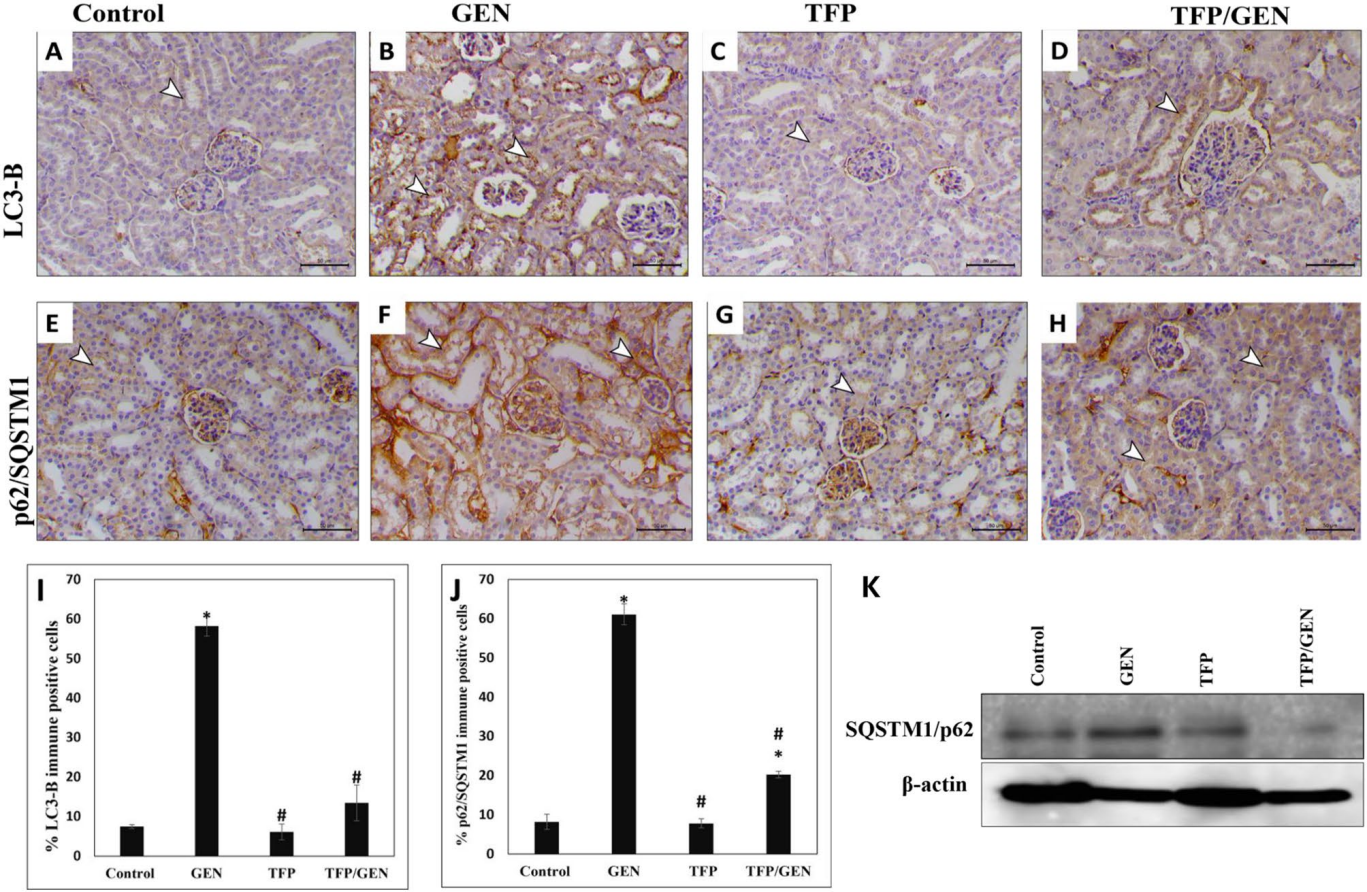
Trifluoperazine alleviated autophagy impairment by gentamicin in the kidneys. Representative photomicrographs of LC3-B and p62/SQSTM1 immunohistochemistry from different groups (n = 5). (**A**) Control LC3-B, (**B**) Gentamicin LC3-B, (**C**) Trifluoperazine LC3-B (**D**) Trifluoperazine/Gentamicin LC3-B, (**E**) Control p62/SQSTM1, (**F**) Gentamicin p62/SQSTM1, (**G**) Trifluoperazine p62/SQSTM1, and (**H**) Trifluoperazine/Gentamicin p62/SQSTM1. *Arrowheads:* positively stained renal cells. Magnification × 200, bar = 50 µm. (**I**) Quantitation of the percentage of LC3-B immunopositive cells in panels A, B, C & D. (**J**) Quantitation of the percentage of p62/SQSTM1 immunopositive cells in panels E, F, G & H. Data are presented as mean ± SD of positively stained cells in 5 high-power fields. ^*^ Denotes statistically significant differences from control group. ^#^ Denotes statistically significant differences from gentamicin group. (**K**) A representative image of Western blotting of p62/SQSTM1 protein expression relative to beta actin loading control in kidney tissues of different groups.

Next, we investigated the downstream signaling cascades of oxidative stress. Autophagy inhibition is a key factor in the pathogenesis of AKI^43^. Accordingly, we assessed the possible autophagy perturbation through immunohistochemical analysis of key markers, LC3-B and p62/SQSTM1^44^. In both the control (Fig. 4A) and trifluoperazine monotherapy (Fig. 4C) groups, LC3-B expression was basal, with around 7% cell positivity (Fig. 4I). In contrast, the gentamicin group showed a marked increase in LC3-B expression, with 58% cell positivity (Fig. 4B and I). Remarkably, pretreatment with trifluoperazine before gentamicin reduced LC3-B positivity to 13.5%, indicating a strong abrogation of gentamicin-induced LC3-B expression (Fig. 4D and I).

Regarding p62/SQSTM1, the control and trifluoperazine groups showed normal expression, with an average of 8% cell positivity (Fig. 4E, G, J). Conversely, gentamicin caused a significant increase in p62/SQSTM1 protein expression level, with 61% cell positivity (Fig. 4F and J). Importantly, administration of trifluoperazine prior to gentamicin treatment decreased p62/SQSTM1 positivity to 20% in treated mice (Fig. 4H and J). Western blot analysis confirmed the immunohistochemistry results, where gentamicin induced upregulation of SQSTM1/p62 was markedly overcome by pretreatment with trifluoperazine (Fig. 4K).

Collectively, these findings indicated that trifluoperazine did not interfere with basal autophagy but effectively preserved autophagy against gentamicin-induced inhibition.

### Trifluoperazine suppresses gentamicin-induced NF-κB signaling

**Fig. 5:**
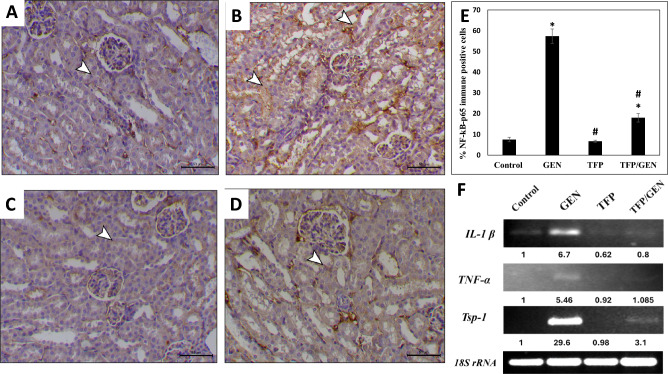
Trifluoperazine hindered gentamicin-induced renal NF-κB-p65 expression and activation. Representative photomicrographs of NF-κB-p65 immunohistochemistry from different groups (n = 5). (**A**) Control, (**B**) Gentamicin, (**C**) Trifluoperazine, (**D**) Trifluoperazine/Gentamicin. *Arrowheads*: positively stained renal cells. Magnification × 200, bar = 50 µm. (**E**) Quantitation of the percentage of NF-κB-p65 immunopositive cells in panels A, B, C, & D. Data are presented as mean ± SD of positively stained cells in 5 high-power fields. * Denotes statistically significant differences from the control group. # Denotes statistically significant differences from the gentamicin group. (**F**) Representative agarose gel electrophoresis and band intensities for PCR analysis of mRNA expression levels of *IL-1β, TNF-α*, *Tsp-1*, and *18S rRNA* housekeeping control.

The crosstalk between oxidative stress and NF-κB is well-established in nephrotoxin-induced AKI^45^. Therefore, to assess NF-κB activation, we measured NF-κB-p65 expression by immunohistochemistry. Results revealed that both control (Fig. 5A and E) and trifluoperazine monotherapy-treated mice (Fig. 5C and E) had similar NF-κB-p65 levels. However, gentamicin treatment notably upregulated NF-κB-p65 expression, with 57% positive staining (Fig. 5B and E). Notably, trifluoperazine pretreatment suppressed NF-κB-p65 expression, with only 18% of cells showing positive staining (Fig. 5D and E).

To validate the immunohistochemistry findings suggesting NF-κB inhibition by trifluoperazine (Fig. 5E), we measured the expression of established NF-κB target genes, interleukin-1β (*IL-1β*), tumor necrosis factor-alpha (*TNF-α*), and thrombospondin-1 (*Tsp-1*). Of note, *IL-1β* and *TNF-α* are pro-inflammatory mediators implicated in gentamicin-induced AKI, while *Tsp-1* is a pro-fibrotic marker upregulated following renal injury that promotes progression to chronic kidney disease^46–48^.

Gentamicin treatment increased *IL-1β* mRNA levels by approximately sevenfold compared to control (Fig. 5F). This upregulation was suppressed by the trifluoperazine pretreatment prior to gentamicin, reducing *IL-1β* expression by 88%, relative to gentamicin group (Fig. 5F). Additionally, trifluoperazine alone reduced *IL-1β* expression by 38% compared to control (Fig. 5F). Similarly, *TNF-α* expression was approximately fivefold higher in the gentamicin group compared to the control group, while trifluoperazine pretreatment reduced it by 80% (Fig. 5F). *TNF-α* levels in the trifluoperazine monotherapy group were comparable to control (Fig. 5F).

*Tsp-1* expression showed approximately a 30-fold increase in the gentamicin group compared to the control, which was reduced by 89% with trifluoperazine pretreatment (Fig. 5F). Expression in the trifluoperazine group remained at control levels (Fig. 5F). Therefore, these findings further indicate that gentamicin induces renal tubular damage. Taken together, these results confirm that trifluoperazine significantly attenuates gentamicin-induced NF-κB activation and its downstream inflammatory and pro-fibrotic gene expression.

### Trifluoperazine ameliorates gentamicin-induced ERK1/2 activation

**Fig. 6:**
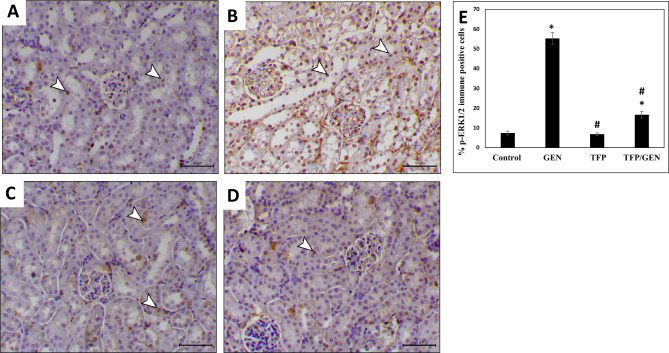
Trifluoperazine suppressed renal ERK1/2 phosphorylation induced by gentamicin. Representative photomicrographs of phospho-ERK1/2 immunohistochemistry from different groups (n = 5). (**A**) Control, (**B**) Gentamicin, (**C**) Trifluoperazine, (**D**) Trifluoperazine/Gentamicin. *Arrowheads:* positively stained renal cells. Magnification × 200, bar = 50 µm. (**E**) Quantitation of the percentage of p-ERK1/2 immunopositive cells in panels A, B, C, & D. Data are presented as mean ± SD of positively stained cells in 5 high-power fields. ^*^ Denotes statistically significant differences from the control group. ^#^ Denotes statistically significant differences from the gentamicin group.

The ERK1/2 pathway is a key mediator in the development of gentamicin-induced AKI^49^. To assess ERK1/2 activation, we evaluated its phosphorylation status via immunohistochemistry. Gentamicin treatment markedly increased ERK1/2 phosphorylation, showing nearly an eightfold elevation compared to the control group (Fig. 6B and E). In comparison, the trifluoperazine monotherapy group displayed phosphorylation levels comparable to the control (Fig. 6C and E). Importantly, in the trifluoperazine/gentamicin group, p-ERK1/2 positive staining was only twofold higher than the control group (Fig. 6D and E). These findings suggest that trifluoperazine efficiently downregulates gentamicin-induced ERK1/2 signaling.

### Trifluoperazine impeded gentamicin-induced activation of NLRP3 inflammasomes

**Fig. 7:**
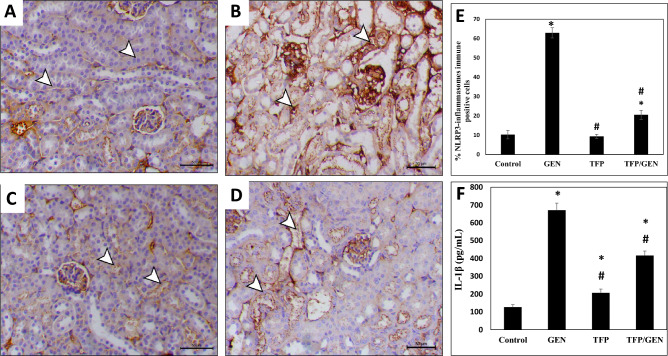
Trifluoperazine reduced gentamicin-induced renal NLRP3 inflammasomes expression and signaling. Representative photomicrographs of NLRP3 inflammasomes immunohistochemistry from different groups (n = 5). (**A**) Control, (**B**) Gentamicin, (**C**) Trifluoperazine, (**D**) Trifluoperazine/Gentamicin. *Arrowheads*: positively stained renal cells. Magnification × 200, bar = 50 µm. (**E**) Quantitation of the percentage of NLRP3 inflammasomes immunopositive cells in panels A, B, C, & D. Data are presented as mean ± SD of positively stained cells in 5 high-power fields. * Denotes statistically significant differences from control group. # Denotes statistically significant differences from gentamicin group. (**F**) ELISA assay of IL-1β content in the kidney tissue homogenates of different groups. Data are presented as mean ± SD (n = 3).

Since activation of NLRP3 inflammasomes promotes gentamicin-induced kidney impairment and renal inflammatory cell death, targeting this pathway may represent a potential therapeutic strategy^50^. To evaluate the effect of treatments on this key signaling event, we assessed NLRP3 expression via immunohistochemistry. Gentamicin treatment markedly increased NLRP3 expression by sixfold compared to the control group (Fig. 7B and E), while the trifluoperazine monotherapy group exhibited expression levels comparable to the control (Fig. 7C and E). Notably, the trifluoperazine/gentamicin group showed only a twofold increase compared to the control (Fig. 7D and E).

Activation of NLRP3 inflammasome signaling ultimately culminates in the production of the pro-inflammatory marker IL-1β^51^ Therefore, to evaluate the impact of the drug treatments on this pathway, we measured IL-1β protein levels in the kidneys. Results revealed that gentamicin significantly increased IL-1β levels by approximately 5.3-fold compared to control (Fig. 7F). Of note, the trifluoperazine pretreatment prior to gentamicin demonstrated a more substantial suppressive effect, reducing IL-1β levels by 62% compared to gentamicin alone (Fig. 7F). These findings indicate that trifluoperazine effectively inhibits gentamicin-induced NLRP3 inflammasomes activation, highlighting its potential to protect against gentamicin-induced pyroptosis in the kidneys.

### Trifluoperazine mitigated gentamicin-induced apoptosis

Since gentamicin-induced AKI eventually triggers renal cell death through multiple pathways^52^, and NLRP3 inflammasome activation is implicated in various forms of cell death in the kidney^51^, we investigated apoptosis by quantifying transformation-related protein 53 (*Trp53)* mRNA expression, which encodes the p53 protein, a master regulator of apoptosis, and by performing caspase-3 immunostaining^53,54^.

**Fig. 8:**
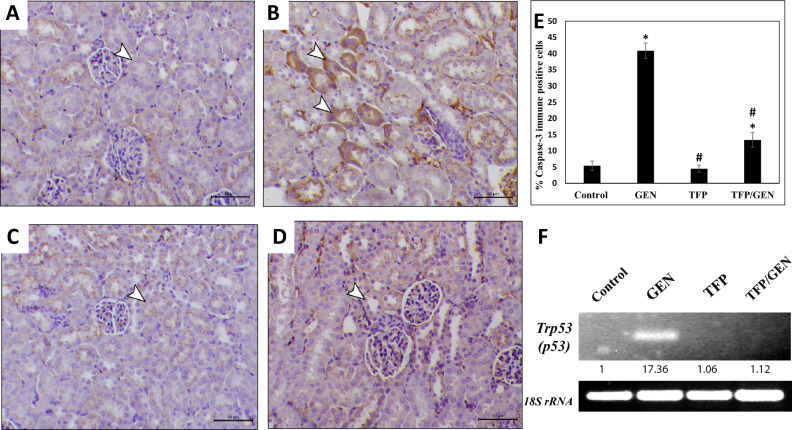
Trifluoperazine overcame gentamicin-induced caspase-3 expression in the kidney. Representative photomicrographs of caspase-3 immunohistochemistry from different groups (n = 5). (**A**) Control, (**B**) Gentamicin, (**C**) Trifluoperazine, (**D**) Trifluoperazine/Gentamicin. *Arrowheads:* positively stained renal cells. Magnification × 200, bar = 50 µm. (**E**) Quantitation of the percentage of caspase-3 immunopositive cells in panels A, B, C, & D. Data are presented as mean ± SD of positively stained cells in 5 high-power fields. ^*^ Denotes statistically significant differences from the control group. ^#^ Denotes statistically significant differences from the gentamicin group. (**F**) Representative agarose gel electrophoresis and band intensities for PCR analysis of mRNA expression levels of *Trp53* and *18S* rRNA housekeeping control.

Our data show that gentamicin significantly increased *Trp53* mRNA expression by approximately 17-fold relative to the control (Fig. 8F). Trifluoperazine alone did not increase *Trp53* levels, whereas pretreatment with trifluoperazine reduced *Trp53* expression by about 93% compared to gentamicin alone (Fig. 8F). Furthermore, caspase-3 immunostaining revealed similar levels between the trifluoperazine and control groups (5.4% vs. 4.5%, respectively) (Fig. 8A, C, and E). In contrast, gentamicin treatment increased caspase-3 positivity to 41% (Fig. 8B and E). Interestingly, trifluoperazine pretreatment attenuated this effect, reducing caspase-3-positive cells to 13% (Fig. 8D and E). These findings suggest that trifluoperazine significantly mitigates gentamicin-induced renal apoptosis.

## Discussion

This study provides the first in vivo preclinical evidence that trifluoperazine pretreatment extends the safe gentamicin treatment period to 8 days, reducing the risk of AKI while preserving kidney structure and function. Previous reports showed that trifluoperazine enhanced antibiotic efficacy against bacterial persister cells by disrupting bacterial metabolism, transcription, translation, and membrane integrity^3,55,56^, and it also inhibited cytokine storms while improving survival in sepsis models^57,58^. These properties, combined with our findings, highlight the potential clinical value of this novel strategy, delivering both renoprotective and antimicrobial benefits.

Oxidative stress plays a central role in gentamicin-induced nephrotoxicity^12^, and our results show that gentamicin depletes renal antioxidant defenses, aligning with previous research^7,11,12^. Trifluoperazine partially ameliorated the impairment of these defenses, likely by dysregulation of the feedback loop between oxidative stress and the mitochondrial permeability transition pore (mPTP)^59^, taking into account that trifluoperazine is a well-known inhibitor of the mPTP^60^.

Beyond causing direct damage, oxidative stress impairs autophagy, worsening AKI^43^ through mechanisms such as oxidation of autophagic proteins, reduced degradation of damaged organelles, and activation of Akt/mTOR signaling^43,61,62^. Maintaining autophagy has been shown to be protective in kidney disease models^43^. In this study, trifluoperazine alleviated gentamicin-induced impairment of autophagy, consistent with its previously reported ability to sustain autophagy in neuronal cells^16,63^. Oxidative stress also triggers NF-κB activation, which drives inflammation and cell death in AKI^45^, with elevated NF-κB activity linked to more severe renal damage^45,50^. Trifluoperazine inhibited NF-κB across various models^22,64,65^, an effect we extended to gentamicin-induced AKI, where its suppression of ERK1/2 phosphorylation, known to enhance NF-κB signaling^66^, likely contributed to this inhibition.

Additionally, gentamicin activates the NLRP3 inflammasome, promoting renal fibrosis^50^, and to our knowledge, this is the first study to demonstrate that trifluoperazine inhibits NLRP3 activation, likely through multiple pathways, including reduced oxidative stress, maintenance of autophagy, NF-κB inhibition, and ERK1/2 suppression^66–71^. Since NLRP3 activation triggers pyroptosis and apoptosis^51^, trifluoperazine’s inhibition of the inflammasome likely reduced both processes in this model.

**Fig. 9:**
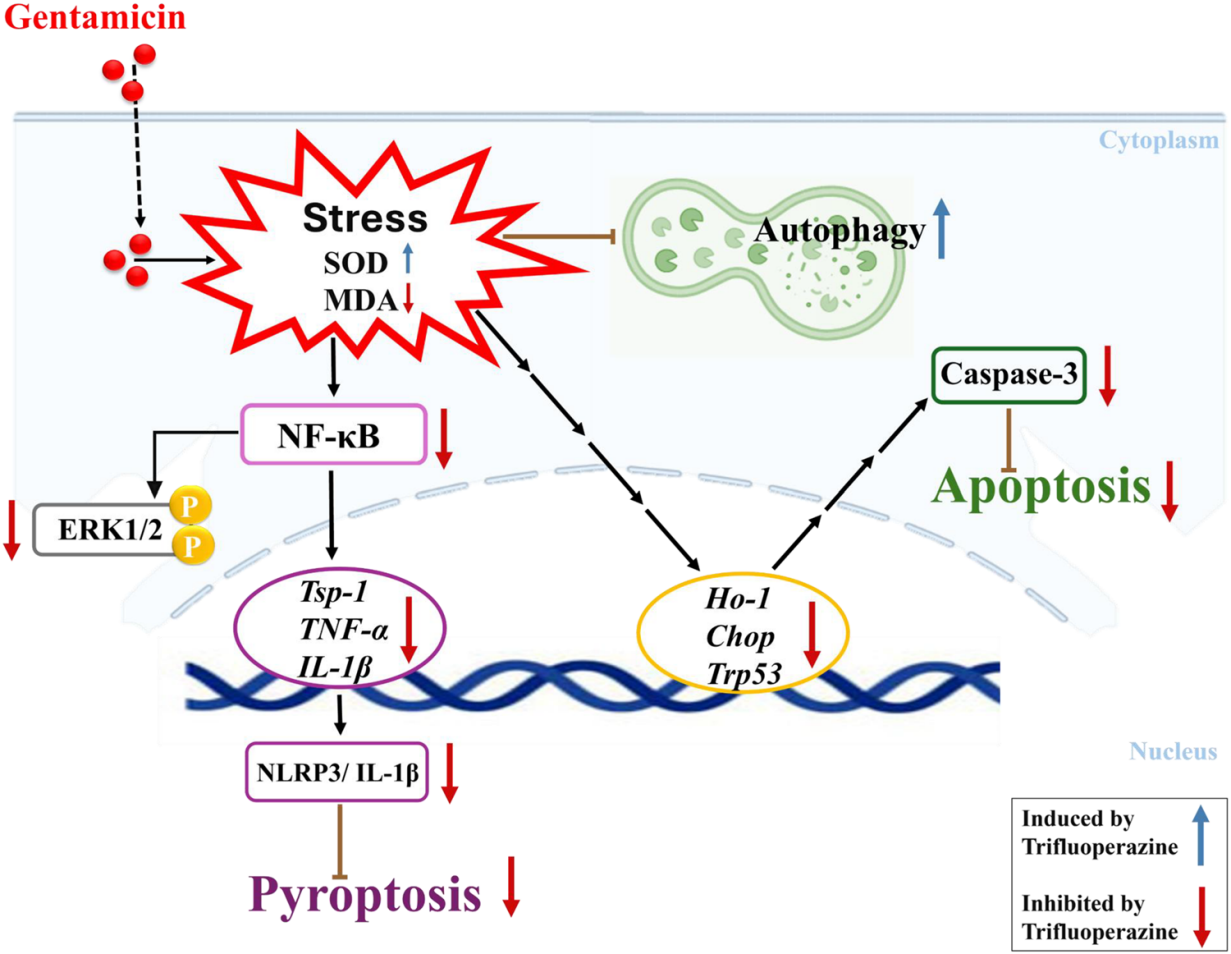
Signaling pathways of trifluoperazine’s protective effect against gentamicin-induced AKI. Graphical summary illustrating the potential signaling pathways involved in the ameliorative effects of trifluoperazine against gentamicin-induced AKI in mice. Red arrows: inhibited by trifluoperazine; Blue arrows: induced by trifluoperazine. SOD, Superoxide Dismutase; MDA, Malondialdehyde; Chop, C/EBP Homologous Protein; HO-1, Heme Oxygenase-1; p-ERK1/2, Phosphorylated extracellular signal-regulated kinase 1/2; NF-κB, Nuclear Factor Kappa B; Tsp-1, Thrombospondin-1; NLRP3, NOD-like receptor family, pyrin domain containing 3; IL-1β, Interleukin-1 beta; TNF-α, Tumor Necrosis Factor-alpha; Trp53, Transformation-related protein 53.

Collectively, the present study provides preclinical evidence that trifluoperazine mitigates gentamicin-induced AKI through multitarget modulation of oxidative stress, autophagy, NF-κB, ERK1/2, NLRP3 inflammasome activation, and apoptosis (Fig. 9). Beyond demonstrating novelty in aminoglycoside-induced nephrotoxicity, our findings support the potential repurposing of trifluoperazine as an adjunctive strategy to reduce drug-induced kidney injury, particularly in patients requiring prolonged aminoglycoside therapy or those at high risk of nephrotoxicity. Moreover, the mechanistic pathways identified suggest broader applicability in other forms of inflammatory- and oxidative stress–mediated AKI (Fig. 9).

A limitation of this study is the absence of direct investigation into the role of calcium/calmodulin-dependent protein kinase II (CaMKII) signaling in the protective effects of trifluoperazine against gentamicin-induced AKI. Recently, CaMKII has emerged as a promising therapeutic target for AKI, with increased expression and/or activation of its isoforms linked to renal injury and remodeling^72^. The calcium/calmodulin/CaMKII pathway is known to modulate oxidative stress and inflammation, key contributors to gentamicin-induced nephrotoxicity^73,74^. Given trifluoperazine’s well-established calmodulin antagonist activity, it is plausible that its observed renoprotective effects may involve this pathway. Previous studies have demonstrated that the calcium/calmodulin/CaMKII axis orchestrates signaling through NF-κB, ERK1/2, and NLRP3 inflammasomes^75–78^, all of which were modulated by trifluoperazine in our study. Furthermore, the suppression of ERK1/2 phosphorylation by trifluoperazine has been previously attributed to its strong antagonistic effect on calcium/calmodulin signaling^79^. However, without direct assessment of the calcium/calmodulin/CaMKII pathway, its involvement in the protective mechanism of trifluoperazine remains speculative. This gap highlights the need for further investigation to confirm whether this pathway mediates the observed effects.

Furthermore, future studies should incorporate complementary experimental approaches, larger sample sizes, and additional animal strains to improve reproducibility and the robustness of the findings. In addition, well-designed translational investigations are warranted to determine the clinical relevance of trifluoperazine in human gentamicin-induced AKI and to evaluate its safety and therapeutic potential in clinically relevant models of renal injury.

In conclusion, our findings suggest that trifluoperazine exerts potential renoprotective effects in gentamicin-induced AKI through multitargeted mechanisms. These results provide a foundation for further exploration, particularly regarding drug repurposing. More comprehensive and translational studies are essential to establish trifluoperazine’s therapeutic potential in clinical settings.

## Supplementary Information

Supplementary Information 1.

Supplementary Information 2.

## Data Availability

All data generated or analysed during this study are included in this published article [and its supplementary information files].
