# Supplementary figures and images for: Amelioration of gentamicin-induced acute kidney injury by trifluoperazine: in vivo mechanistic insights
Source: Sci Rep. 2026 Apr 20;16:12896. doi: 10.1038/s41598-026-47243-w (PMC13096156; doi:10.1038/s41598-026-47243-w)

**18s rRNA**

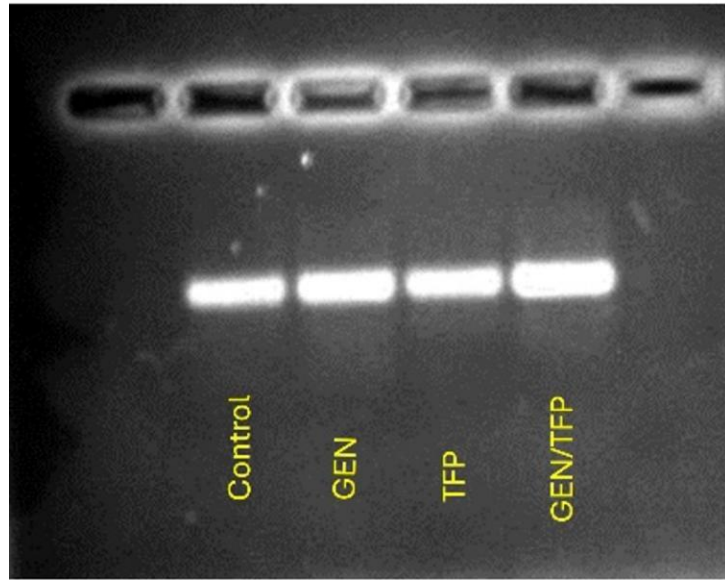

**TSP1**

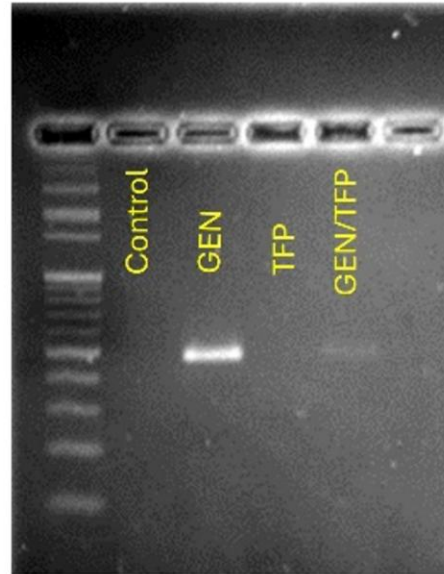

**CHOP**

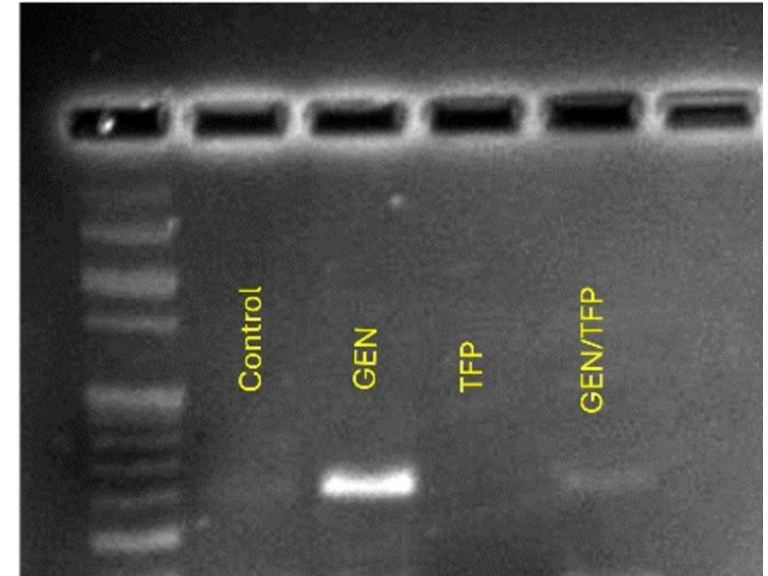

**HO-1**

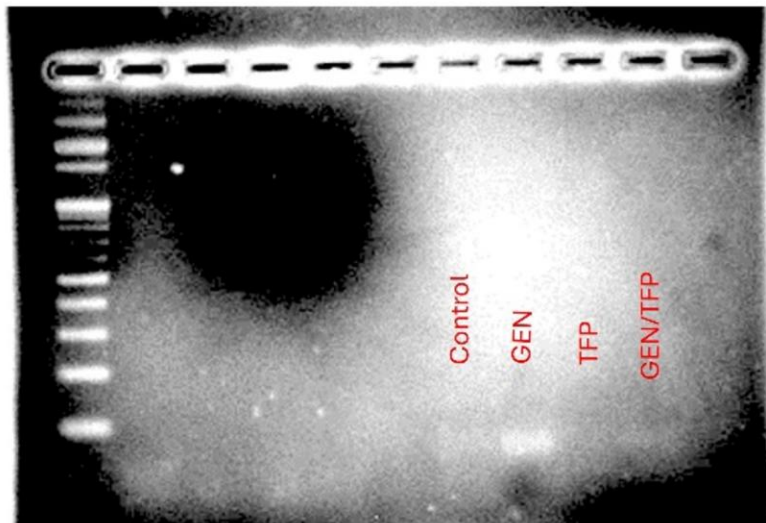

**IL Beta**

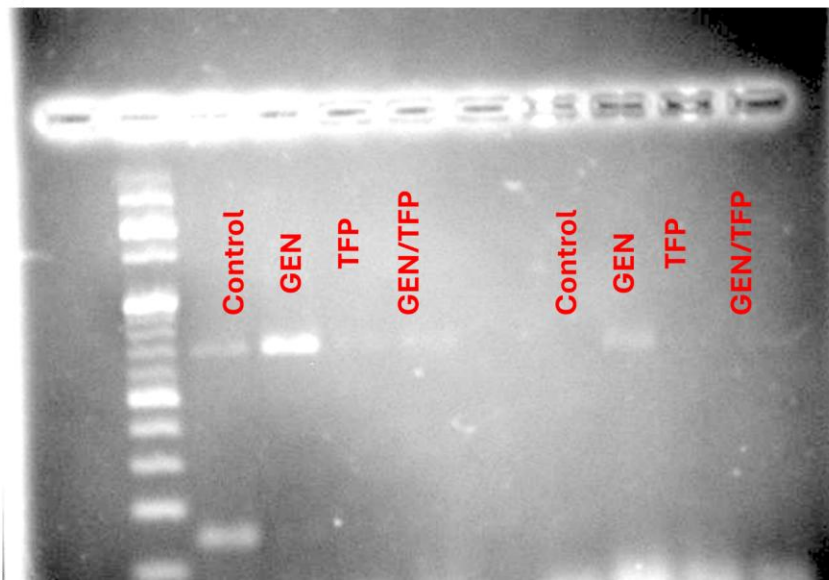

**TNF alpha**

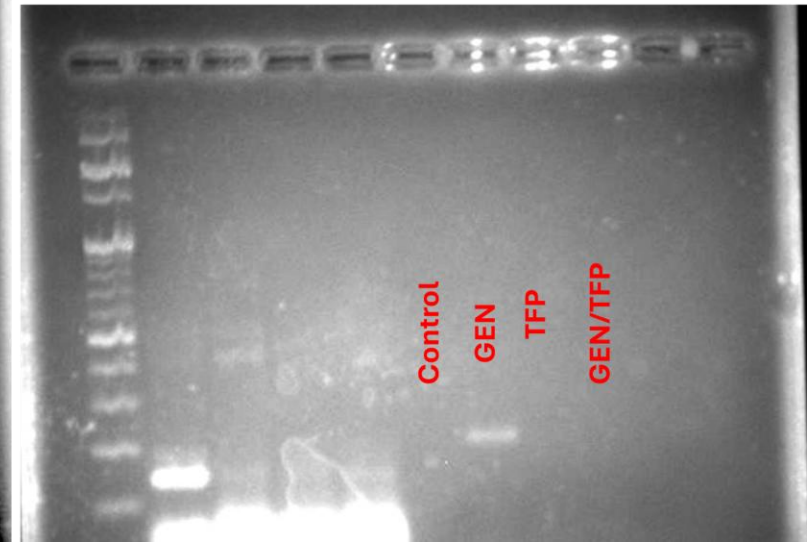

**Trp53  
(p53)**

Supplement: Supplementary file 1 — Supplementary Information 1. [file 41598_2026_47243_MOESM1_ESM.pdf]
