# Supplementary material for: Amelioration of gentamicin-induced acute kidney injury by trifluoperazine: in vivo mechanistic insights
Source: Sci Rep. 2026 Apr 20;16:12896. doi: 10.1038/s41598-026-47243-w (PMC13096156; doi:10.1038/s41598-026-47243-w)
Supplement: Supplementary file 2 — Supplementary Information 2. [file 41598_2026_47243_MOESM2_ESM.pdf]

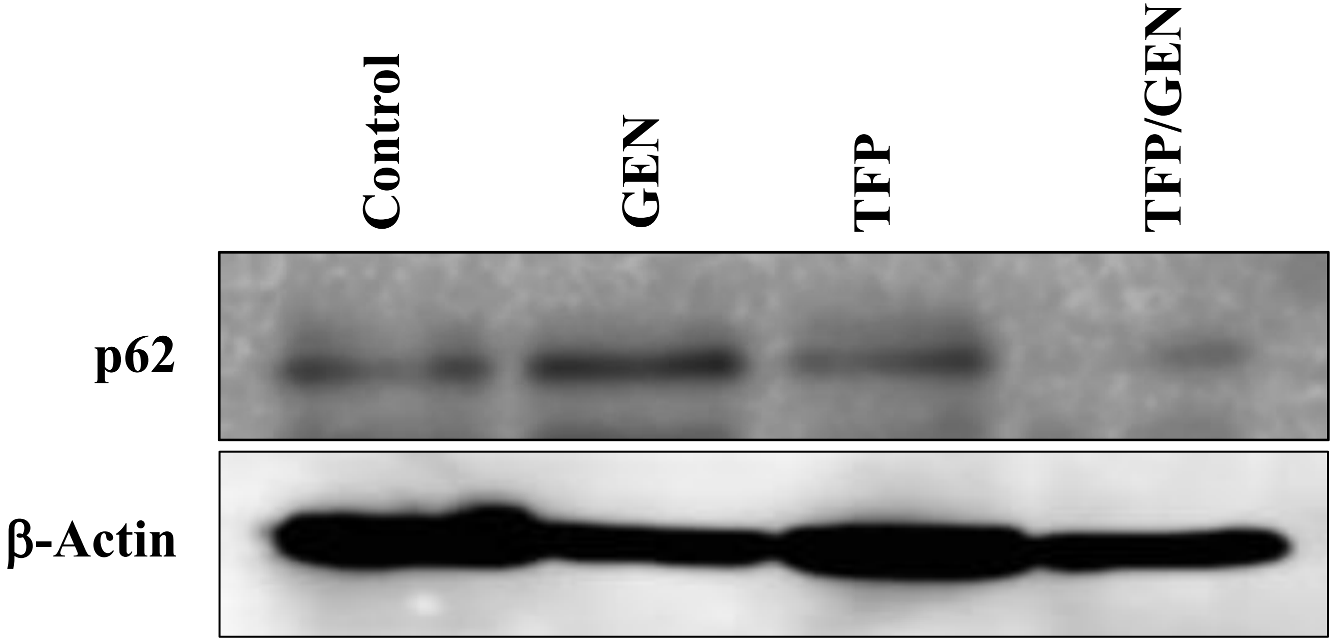
Control
GEN
TFP
TFP/GEN
p62
β-Actin

Different exposures of p62

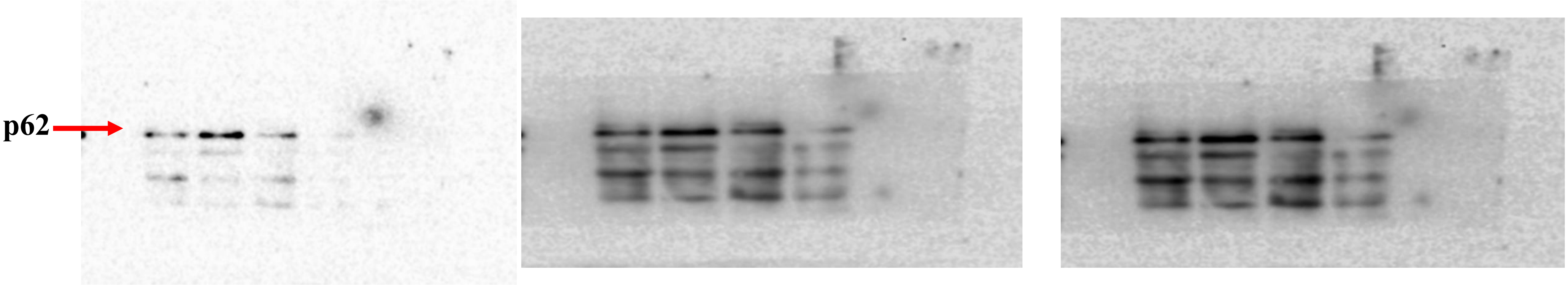

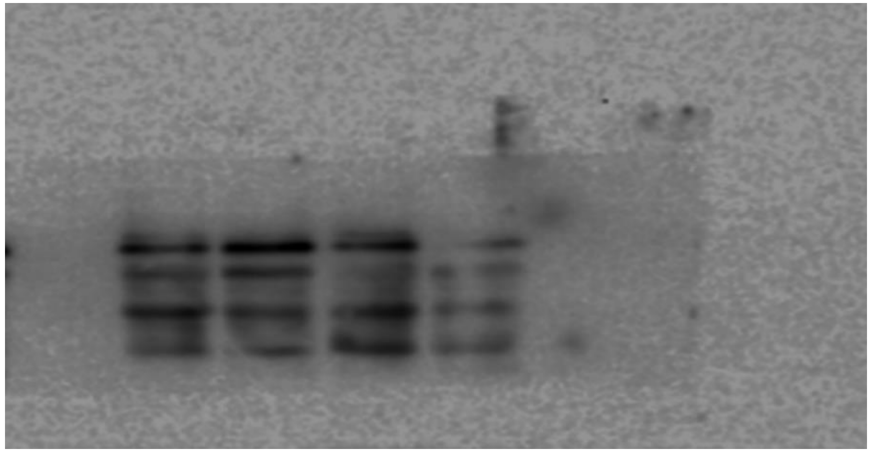

Membrane ECL images

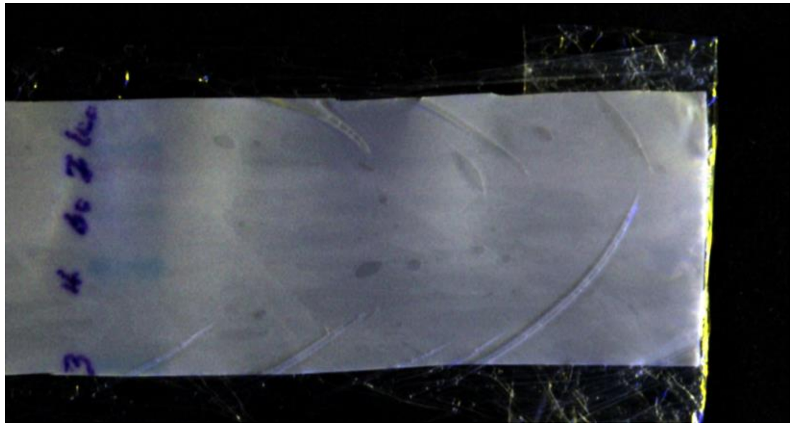

membrane LED image

Different exposures of beta Actin

β-Actin →

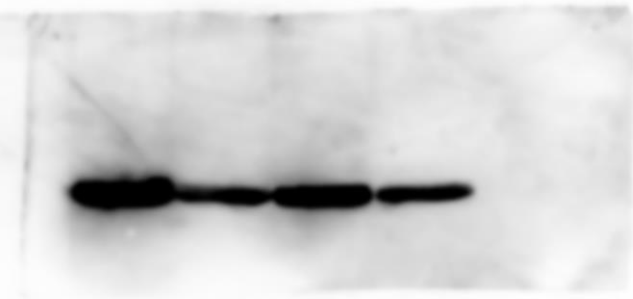

Membrane ECL image

β-Actin →

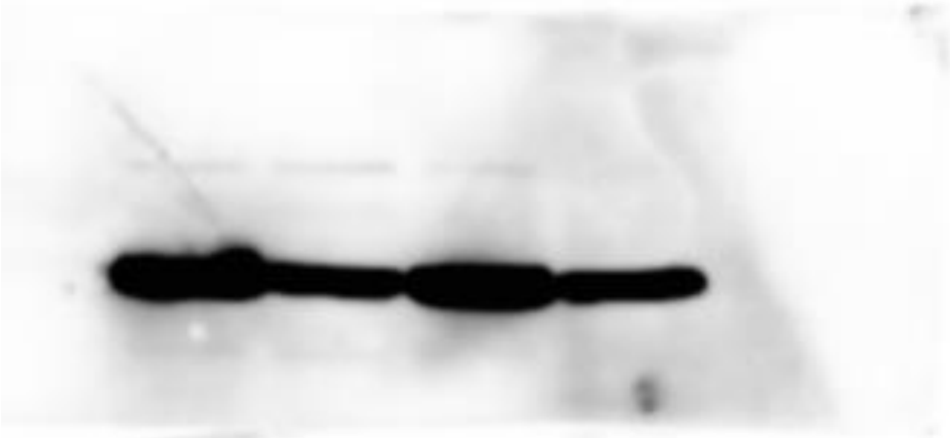

Membrane ECL image

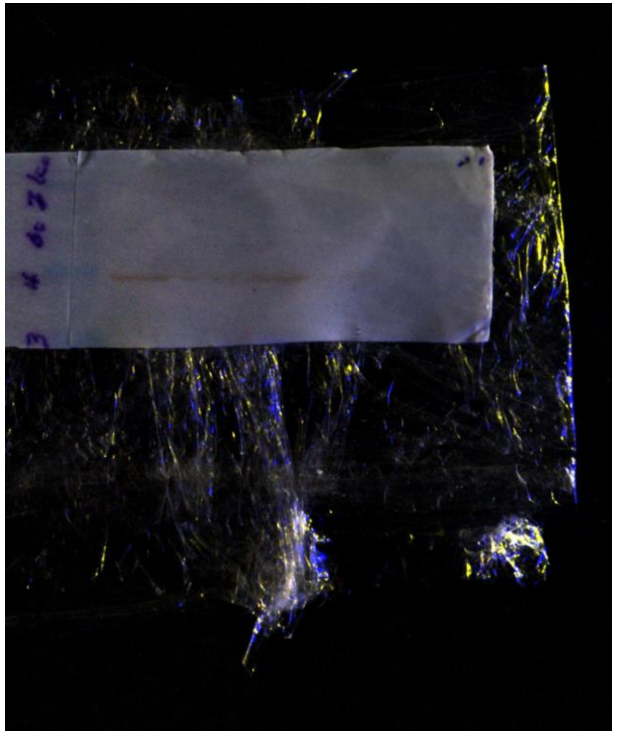

membrane LED image

Automatic overlay by Azure software for ECL and LED membrane images →

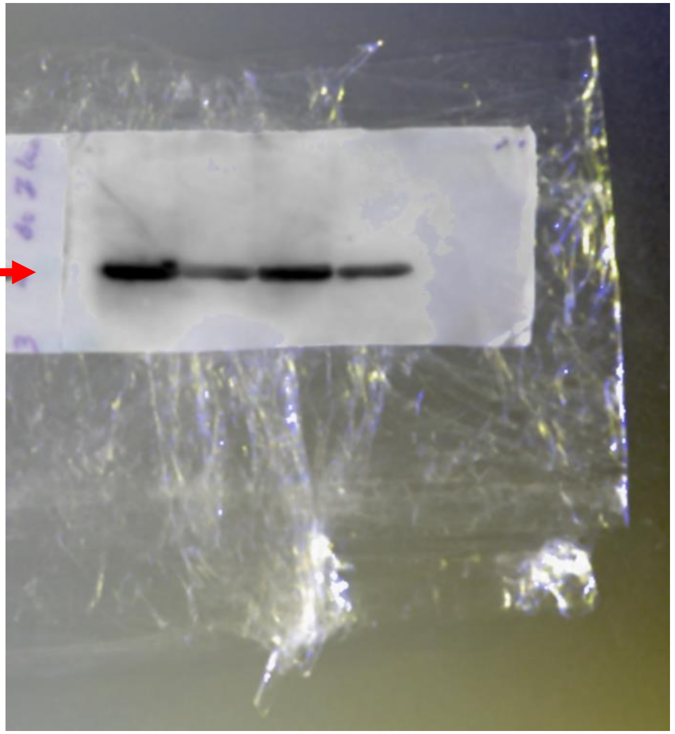

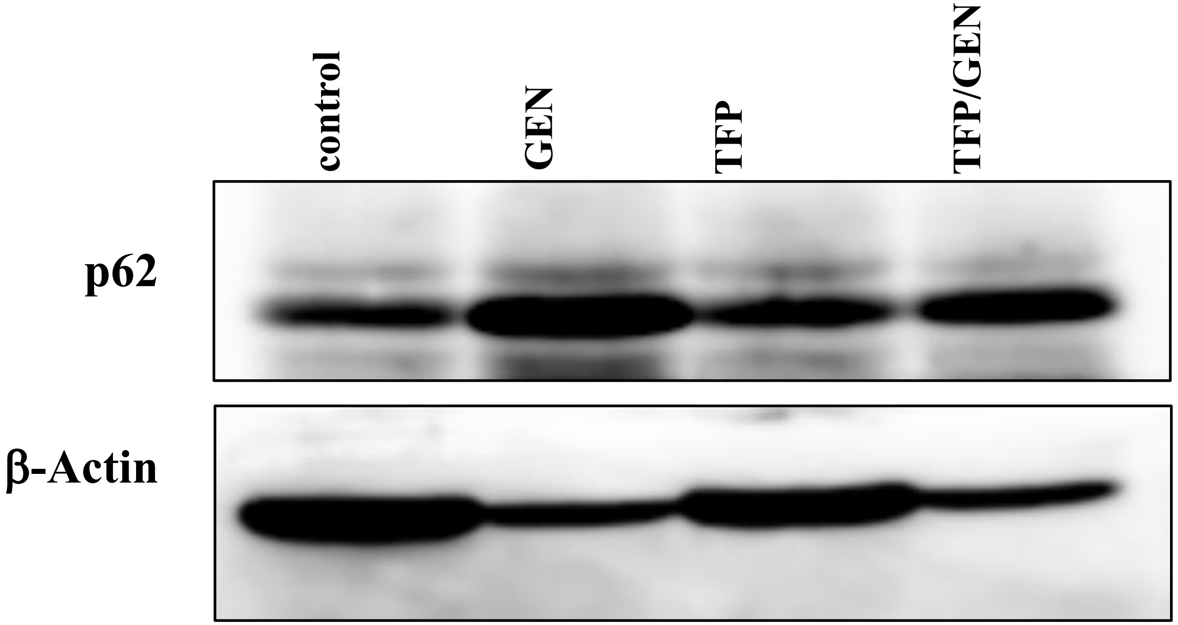
control
GEN
TFP
TFP/GEN
p62
β-Actin

Membrane ECL image

p62

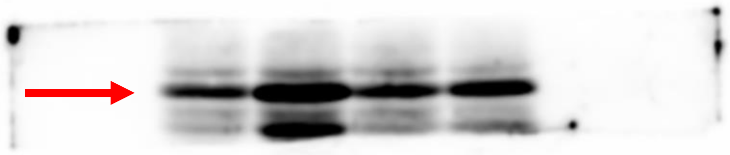

Membrane LED image

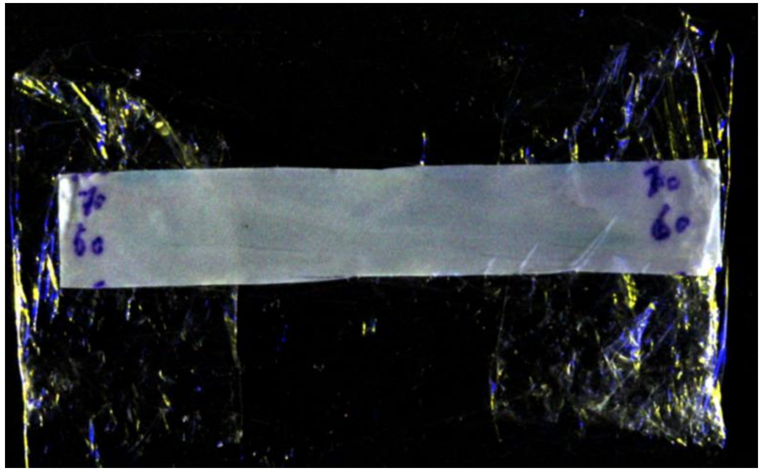

Automatic overlay by Azure software for ECL and LED membrane images

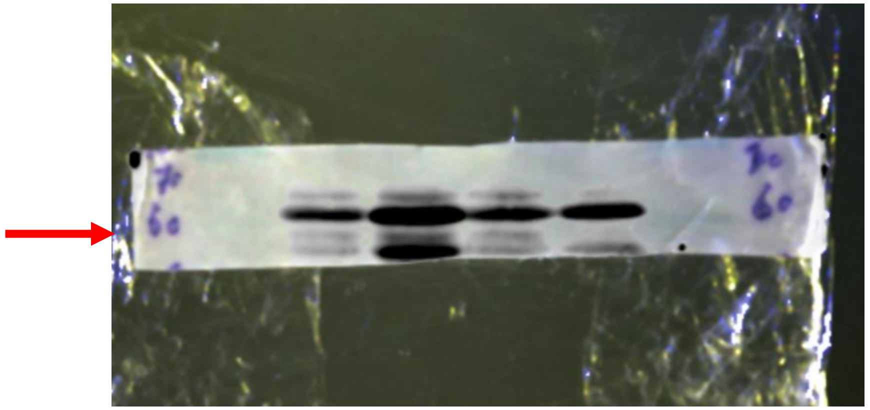

β-Actin

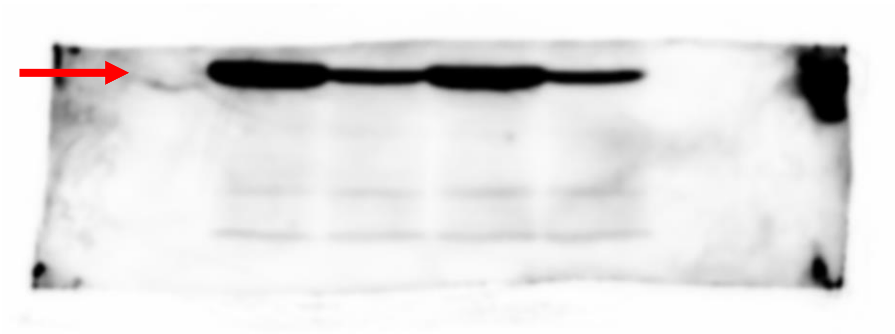

Membrane ECL image

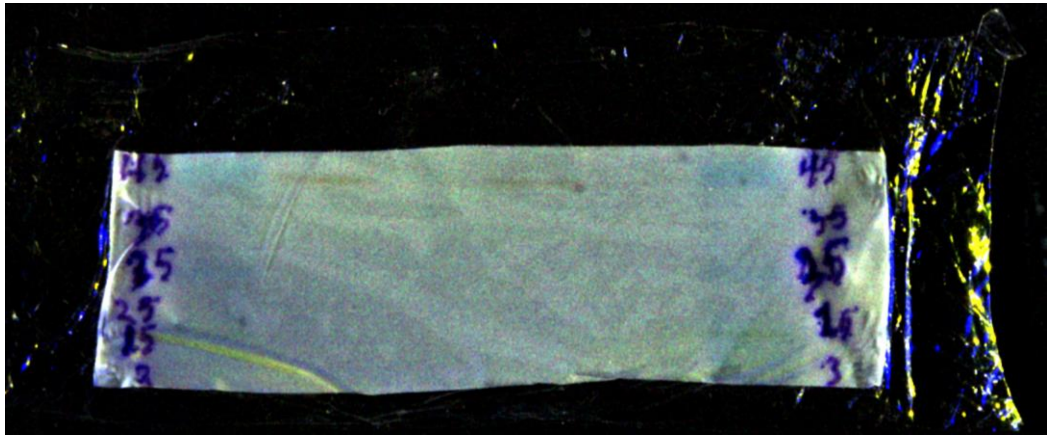

membrane LED image

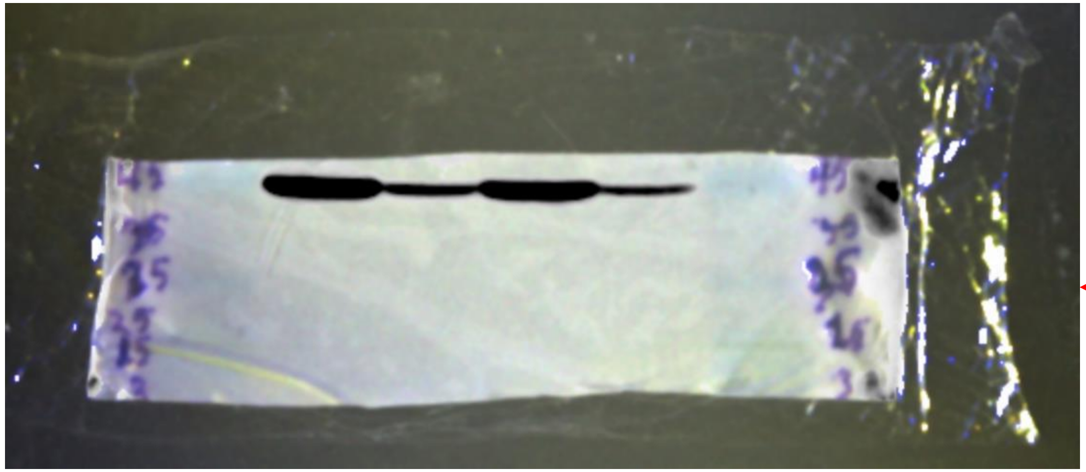

Automatic overlay by Azure software for ECL and membrane LED images
